# Insights into the Life Cycle of Yeasts from the CTG Clade Revealed by the Analysis of the *Millerozyma (Pichia) farinosa* Species Complex

**DOI:** 10.1371/journal.pone.0035842

**Published:** 2012-05-04

**Authors:** Sandrine Mallet, Stéphanie Weiss, Noémie Jacques, Véronique Leh-Louis, Christine Sacerdot, Serge Casaregola

**Affiliations:** 1 INRA UMR1319, Micalis Institute, CIRM-Levures, Thiverval-Grignon, France; 2 AgroParisTech UMR1319, Micalis Institute, Thiverval-Grignon, France; 3 Université de Strasbourg UMR7156, CNRS, Strasbourg, France; 4 Institut Pasteur, Unité de Génétique Moléculaire des Levures, CNRS, UMR 3525, Université Pierre et Marie Curie-Paris 06, UFR 927, Paris, France; Belgian Nuclear Research Centre SCK/CEN, Belgium

## Abstract

Among ascomycetous yeasts, the CTG clade is so-called because its constituent species translate CTG as serine instead of leucine. Though the biology of certain pathogenic species such as *Candida albicans* has been much studied, little is known about the life cycles of non-pathogen species of the CTG clade. Taking advantage of the recently obtained sequence of the biotechnological *Millerozyma* (*Pichiasorbitophila*) *farinosa* strain CBS 7064, we used MLST to better define phylogenic relationships between most of the *Millerozyma farinosa* strains available in public collections. This led to the constitution of four phylogenetic clades diverging from 8% to 15% at the DNA level and possibly constituting a species complex (*M. farinosa*) and to the proposal of two new species:*Millerozyma miso* sp. nov. CBS 2004^T^ ( = CLIB 1230^T^) and *Candida pseudofarinosa* sp. nov.NCYC 386^T^( = CLIB 1231^T^).Further analysis showed that *M. farinosa* isolates exist as haploid and inter-clade hybrids. Despite the sequence divergence between the clades, secondary contacts after reproductive isolation were evidenced, as revealed by both introgression and mitochondria transfer between clades. We also showed that the inter-clade hybrids do sporulate to generate mainly viable vegetative diploid spores that are not the result of meiosis, and very rarely aneuploid spores possibly through the loss of heterozygosity during sporulation. Taken together, these results show that in this part of the CTG clade, non-Mendelian genetic exchanges occur at high rates through hybridization between divergent strainsfrom distinct clades and subsequent massive loss of heterozygosity. This combination of mechanisms could constitute an alternative sexuality leading to an unsuspected biodiversity.

## Introduction

The life cycle of *Saccharomyces* species is well known. They propagate as haploid or diploids according to the environment, mate, sporulate and undergo meiosis to yield haploid spores [1]. *Saccharomyces* species are also known for forming inter-specific hybrids under the pressure of industrial fermentation conditions [2]. This genus is nevertheless far from being representative of the ascomycetous yeasts. Some *Candida* sp. pathogens belonging to the CTG clade were shown to have various life cycles. *Candida albicans* is a diploid that does mate and shows parasexuality, but does not perform sporulation or meiosis [3], [4]. *Clavispora lusitaniae*, on the other hand, can mate and sporulate. Its progeny is complex and comprises haploids resulting from meiosis, diploids and aneuploids [5]. *Candida tropicalis* was also recently shown to display parasexuality [6], suggesting that yeast pathogens are sexual species.

However, little is known about non-pathogenic species of this clade like the most studied *Debaryomyces hansenii*. This species is thought to have a rather clonal reproduction through fusion between mother and daughter cells followed by meiosis, whilemating between individual cells israrely observed [7]. Another CTG species, *Pichia farinosa* (Lindner) E.C. Hansen var. *farinosa* (teleomorph of *Candida cacaoi*) is a ubiquitous, halotolerant yeast found mainly in food (alcoholic beverages like beer and sake, soy sauce, miso, mash of rice vinegar etc.) but alsoon various substrates (cows with mastitis, sputum, giraffedung, laboratory media and highly concentrated sorbitol solutions) [8]. A reassessment of the phylogeny of Q9 enzyme-producing species changed the name of *P. farinosa* to *Millerozyma farinosa* [9]. Increased numbers of *M. farinosa* and other yeasts, mainly *Candida albicans*, were found in patients undergoing radiotherapy for cancer of the mouth [10] and an *M. farinosa* strain was found as an oral cavity colonizer [11]. Two further cases of human infections associated to *M. farinosa* were also described [12], [13].

In 1980, a related species, *Pichia sorbitophila,* was described [14]. The only member of this species, CBS 7064, is able to survive extremely high extracellular concentrations of salts (e.g., saturated solution of KCl) and other osmolytes (70% glucitol), although it has not been classified as halophilic or osmophilic [15], [16]. Both *M. farinosa* and *P. sorbitophila* can produce high yields of glycerol and xylitol, which makes them valuable in biotechnology. The placementof some strains in the *M. farinosa* species is a matter of debate [8]. However, the main controversy has concerned the taxonomy of *M. farinosa*and *P. sorbitophila*. On the basis of DNA relatedness, *Debaryomyces halotolerans*, *P. sorbitophila, *and *Pichia petrophilum* were considered to be conspecific with *M. farinosa* [17], whereas physiological properties and sequence analysis indicated they should be classified as separate species,in agreement with the divergent sequences of their respective mitochondrial DNAs [18], [19]. *M. farinosa* CBS 7064 carries two divergent *URA3* genes, which were also different from that of the *M. farinosa* type strain [20]. The presence of the two divergent *URA3* gene copies and the higher number of chromosomes in pulse field gel electrophoresis was in agreement with CBS 7064 being diploid.

From therecently sequenced CBS 7064 genome [21], it appears thatthis strain is a recent hybrid between two distant parents: the two copies of their homeologous chromosomes present more than 15% divergence at the DNA level. The parental contributorsremain unknown, but are certainly distinct from the *M. farinosa* type strain CBS 185^T^. CBS 7064 contains sevenpairs of chromosome. In three ofthese both parents contribute one chromosome; in two the chromosomes contain regions that are identical and regions that are diverged;two consist of identical chromosomes, indicative of massive loss of heterozygosity (LOH)[21]. Our analysis of the respective contribution of each parent to the hybrid reveals the major genetic changes that occurredduring the time that separated the last common ancestor of both parents and the hybridization event. These include one reciprocal translocation between two chromosomes, a number of gene losses and unequal mitochondrial DNA insertions (NUMT) in the nuclear genomes of the parents.

In order to define the parental contributors of CBS 7064, to resolve the taxonomy of the *M. farinosa* species and to gain insight into the life cycle of CTG species, we took advantage of the available complete genome sequence of the CBS 7064 strain [21] to analyzealmost all the *M. farinosa* strains available in public collections. In this paperwe use MLST to constitute phylogenetic groups. We demonstrate reproductive isolation of these groups. We show that *M. farinosa* is a species complex made of distinct taxa, and in which isolates exist as haploid organisms and as hybrids resulting from crosses between strains from these taxa. We also show that the hybrids can sporulate to generate viable diploid spores that are not the result of meiosis. In this species complex, an alternative sexuality allows the formation of hybrids subjected to LOH and of rare aneuploids.

## Results

### Structuring the *Millerozyma farinosa* populations in five clades

**Figure 1:**
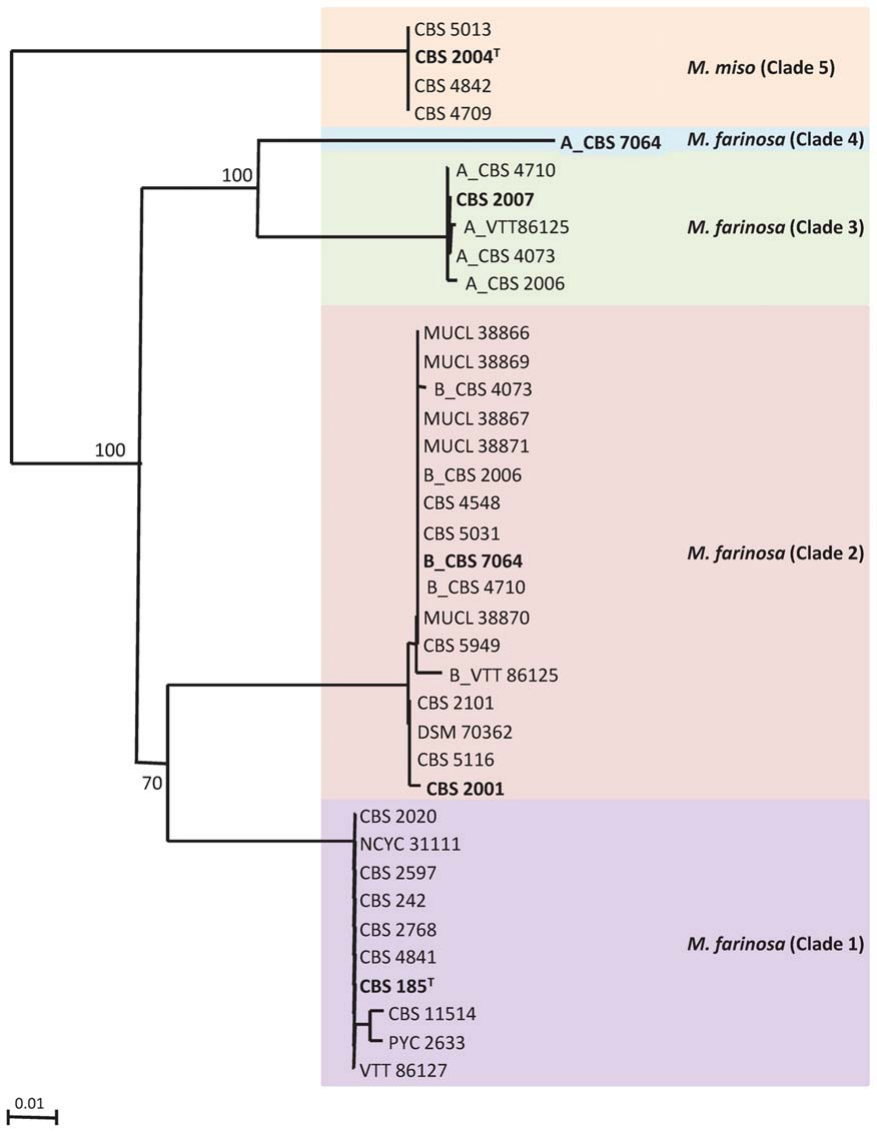
Neighbor-joining phylogram of the *ACT1* gene intron of *M. farinosa* isolates. Bootstrap values (%) based on 1000 replicates are indicated at the nodes for main groups and clades. All positions containing gaps and missing data were eliminated from the dataset. Clades are indicated.Typical strains are in bold characters. Heterozygous alleles in hybrids were arbitrarily given the prefix A or B. Bar, 0.01 substitutions per site.

In a previous study, we have observed that *Debaryomyces hansenii*, a closely related species to *M. farinose* [22], consisted of distinct clades with haploid and diploid strains, the latter being hybrids from diverged strains belonging to these distinct clades [23]. We also demonstrated the efficacy of housekeeping gene intron sequence analysis in distinguishing between clades of *D. hansenii*. The most informative intron sequences were those of the *ACT1* and *RPL33* genes. In order to analyze the *M. farinosa* species, we therefore amplified and sequenced the introns of these two genes in 41 *M. farinosa* strains (Table 1). In a number of cases, PCR products were a mixture of two species which needed to be cloned before sequencing. In these cases, sequences were arbitrarily given the prefix A and B. We compared the obtained sequences and constructed gene trees using the neighbor-joining algorithm. Identical topologies were obtained when the trees were constructed with maximum-likelihood program (data not shown). The *ACT1* gene intron tree is shown in Figure 1 and the *RPL33* gene intron tree is shown in Figure S1. This analysis, both with *ACT1* and *RPL33*, separated the sequences into the same five clades. Some strains displayed two different ACT1 alleles and one RPL33 allele or vice-versa, consistent with LOH. Using flow cytometry, we confirmed that the strains presenting *ACT1* and/or*RPL33* heterozygous alleles had a 2n DNA content. Table 2 gives the estimated genome size of all the strains presenting some heterozygosityfor *ACT 1 *and/or *RPL33* together with ahaploid strain from each clade as a control. For CBS 185^T^ and the haploidstrain of each clade, the estimated size of the genome varied from 9.2 to 10.8 Mb, similar to that of some species in theSaccharomycetaceae [24], but smaller than that of the closely related species *D. hansenii* (13.2 Mb) [25], [26]. The estimated values for the strains presenting sequence heterozygosityvaried between 18 and 22.9 Mb, approximatelytwice the value of CBS 185^T^. An example in Figure S2 shows a shift of the fluorescence intensity peaks in the strains CBS 7064 and CBS 5730 compared to CBS 185^T^ and CBS 2001. These observations strongly suggest that the strains displaying heterozygosity result from crosses between strains belonging to different clades, as already observed for *D. hansenii* [23].

**Table 1:** List of strains used in this study.

Species	Strain number	Substrate of isolation	Country of origin	Ploidy	Clade	Sporulation
*Millerozyma farinosa*	CBS 185^T^	Jopen beer	Poland	haploid	1	+
	CBS 242	katjang-bungil	unknown	haploid	1	nd
	CBS 2020	fermenting cacao	Trinidad and Tobago	haploid	1	nd
	CBS 2597	liquid medium, for tissue culture	UK	haploid	1	nd
	CBS 2768	mash of rice vinegar	Japan	nd	1	nd
	CBS 4841	miso	unknown	nd	1	nd
	CBS 11514	human infection	Israel	nd	1	nd
	NCYC 3111	food industry	UK	nd	1	nd
	NBRC 0602	tamari koji	Japan	nd	1	nd
	PYCC 2633	human	Israel	nd	1	nd
	VTT C-86127	unknown	unknown	nd	1	nd
	CBS 2001^T^	sputum	Norway	haploid	2	−
	CBS 2101	lung of hen	UK	haploid	2	+
	CBS 4548	dung of giraffe	Japan	nd	2	−
	CBS 5031	maize meal	South Africa	nd	2	+
	CBS 5116	mastitis milk	Switzerland	nd	2	−
	DSMZ 70362	mastitis of a cow	unknown	nd	2	+
	MUCL 38866	soy sauce mash (moromi)	Indonesia	nd	2	nd
	MUCL 38867	soy sauce mash (moromi)	Indonesia	nd	2	nd
	MUCL 38869	soy sauce mash (moromi)	Indonesia	nd	2	nd
	MUCL 38870	soy sauce mash (moromi)	Indonesia	nd	2	nd
	MUCL 38871	soy sauce mash (moromi)	Indonesia	nd	2	+
	CBS 2007	sake	unknown	haploid	3	−
	NBRC 10896	unknown	unknown	nd	3	nd
	CBS 7064	70% sorbitol solution	Germany	diploid	2–4	+
	CBS 8045	sorbitol stock	South Africa	diploid	2–4	+
	CBS 2006	homare-miso	unknown	diploid	2–3	+
	CBS 4073	tamari koji	unknown	diploid	2–3	−
	CBS 4710	miso	Japan	diploid	2–3	+
	CBS 5949	soya mash	Japan	diploid	2–3	no growth
	CBS 5730	edo miso	Japan	diploid	2–3	+
	CBS 7911	soy sauce	China	diploid	*−3	+
	VTT C-86125	unknown	unknown	diploid	2–3	
	MUCL 30824	liquid sugar	Belgium	diploid	nd	+
	CLIB 1250	Spore 28 from CBS 2006	−	aneuploid	2–3	nd
	CLIB 1251	Spore G6 from CBS 2006	−	diploid	2–3	nd
*Millerozyma miso*	CBS 2004^T^ (CLIB 1230^T^)	sendai miso	unknown	haploid	5	+
	CBS 4709	miso	Japan	nd	5	nd
	CBS 4842	unknown	unknown	nd	5	nd
	CBS 5013	maize meal	South Africa	nd	5	nd
	NBRC 01003	unknown	unknown	nd	5**	nd
*M. farinosa x M. miso*	CBS 2607	liquid medium for tissue culture	UK	diploid	3–5	+
*Candida pseudofarinosa*	NCYC 386^T^ (CLIB 1231^T^)	unknown	Japan	haploid	na	–
	NBRC 0465	unknown	unknown	nd	na	nd
	NBRC 0574	unknown	unknown	nd	na	nd

nd: not determined, na: not applicable,* 0.5% divergence in *ACT1* with clade 1, ** 1.3% divergence in *ACT1* with clade 4.

For a number of strains, *ACT1* and *RPL33* introns could not be PCR-amplified and the sequence of their *ACT1* coding gene helped to place these strains into the different clades. This is the case for NBRC 0602 and NBRC 10896, CBS 2607(carrying a clade 2 allele and a clade 5 allele and being the only strains carrying this type of combination), NBRC 01003, (** 1.3% divergence with clade 4) and CBS 7911 (a clade 3 allele and *0.5 % divergence with clade 1 allele). MUCL 30824 presented a complex distribution of alleles. The strain CECT 10348 was not related to *M. farinosa*, but rather to the type strain NRRL YB-2798T of *Sugiyamaella* sp. [28] with 2 bp difference over the rDNA D1D2 585 bp long sequence.

**Table 2:** Genome size of various *M. farinose* strains estimated by flow-cytometry.

Strains	Genome size (Mb)	Ratio strain/type strain
CBS 2004^T^	9.20+/−0.2	0.85
CBS 2001	10.6+/−0.2	0.98
CBS 2007	10.6+/−0.5	0.98
CBS 185^T^	10.8+/−0.5	1.00
CBS 5949	18.0+/−0.3	1.67
CBS 7064	18.9+/−0.6	1.75
CBS 5730	20.1+/−1.3	1.86
CBS 8045	20.5+/−0.6	1.90
MUCL 30824	21.1+/−0.9	1.95
CBS 4073	21.3+/−0.3	1.97
CBS 2006	21.9+/−1.5	2.02
CBS 4710	22.4+/−0.5	2.07
CBS 7911	22.9+/−1.4	2.12
CLIB 1251 (spore G6)	24.9+/−0.9	2.30
CLIB 1250 (spore 28)	13.1+/−0.2	1.23

The ratio of the genome size of each strain on that of CBS 185^T^ is indicated.

The first clade (ascribed the number 1) was defined by the sequence of the *ACT1* alleles of 10 strains including the type strain CBS 185^T^. As mentioned above, the results from the analysis of the *RPL33* alleles gave essentially the same clades and are not further discussed here. Clade 2 contained 18sequencesincluding one of the*ACT1* alleles of CBS 7064. Sequence divergence in the *ACT1* intron between these two clades was 8.5% (Table 3). We note that the five strains MUCL 38866, MUCL 38867, MUCL 38869, MUCL 38870 and MUCL 38871 all belong to clade 2 and have identical intronsequences. Since they were all isolated from the same substrate in Indonesia, they may well constitute asingle clone. The clade 3 is comprised of 5 isolates. Only one of these strains, CBS 2007, carried a unique copy of *ACT1* and of *RPL33*; the four other strains, CBS 2006, CBS 4073, CBS 4710 and VTT 86125, each carried two alleles of *ACT1*, one grouping with clade 3 and the other with clade 2. The average sequence divergence in the *ACT1* intronbetween clade 1 and clade 3 was 11.5% (Table 3). The fourth clade contains only one sequence, being that of the second *ACT1* allele of *M. farinosa* CBS 7064, withasequencehighly divergent (13.7%) from that of clade 1 (Table 3). Finally, clade 5 consisted of the sequences of four strains that showed 14.5% divergence fromclade 1 *ACT1* intron sequences and an even higher sequence divergence – above 15% with that of the other clades. Because intronic sequences could not be obtained for anumber of strains, weused coding sequences to place these strainsinto the defined clades (Table 1 and Table S2).

**Table 3:** Sequence divergence (%) within the *ACT1* gene intron between *M. farinosa* clades.

Clades		1	2	3	4	5
	Strains	CBS 185^T^	CBS 2001	CBS 2007	CBS 7064^a^	CBS 2004^ T^
**1**	**CBS** **185^T^**	−	8.5	11.3	13.7	14.5
**2**	**CBS** **2001**		−	11.7	14.5	16
**3**	**CBS** **2007**			−	11	17
**4**	**CBS** **7064** a				−	19.5
**5**	**CBS** **2004^ T^**					−

a Clade 4 allele.

As seen above, sequence divergence between the clades varied from 8.5 to 19.5% (Table 3), indicating that these clades are clearly distinct. We defined a typical strain for each clade as being, among haploids, the first strain described (see the *ACT1* gene intron tree in Figure 1). The topology of the *RPL33* gene intron tree (Figure S1) was very slightly different from that of the *ACT1* gene tree. Comparison of the two gene trees indicated that the clades were composed of the same strains, suggesting the absence of marker exchange between clades, *ACT1* and *RPL33* being located on different chromosomes ([21]; our unpublished data).

Interestingly, one of the two *RPL33* alleles of strain CBS 8045 (the defining and unique strain of clade 4) was different from all sequenced *RPL33* alleles; the other allele belongs to clade 2 as does the unique *ACT1* allele of this hybrid. Since CBS 7064 was homozygous for clade 2 *RPL33* allele ([21]; our data), no “clade 4 *RPL33* sequence” was available. To know whether CBS 8045 was also, like CBS 7064, a clade 2/clade 4 hybrid and effectively carried clade 4 sequences, three markers AB-r, CD-l and IJ-r(see Table S3 and Figure S3) known to existin two heterozygous copies in the genome of CBS 7064 were sequenced in CBS 8045.For each locus we found identical sequences in CBS 7064 and in CBS 8045, indicating that CBS 8045 was indeed another strain carrying both clade 2 and clade 4 sequences. Interestingly, both strains had been isolated as contaminants from highly concentrated sorbitol solutions [27].

Finally, two strains, CECT 10348 and NCYC 386^T^, previously assigned to the *M. farinosa* species did not fall in any of our five clades. CECT 10348 was unrelated to our test group and belonged in fact on the basis of rDNA D1/D2 sequence comparison to a species of the recently described genus *Sugiyamaella* [28]. The strain NCYC 386^T^, though not within the *M. farinosa* complex was the closest on the basis of the *ACT1* coding sequence. Nevertheless, its *ACT1* intron does not show any similarity with that of any strain from the five clades of *M. farinosa* (see later).

### Origin of the CBS 7064chromosomes in relation with the *M. farinosa* clades

Using two housekeeping genes *ACT1* and *RPL33*, we were able to define five distinct clades in the *M. farinosa* species (see above). This MLST analysisshowed unambiguously that strains belonging to clade 2 and clade 4 contributed to the hybrid CBS 7064. In the description of the genome of CBS 7064, a distinction was made between the two parental contributing genomes (designated Pγ and Pε?) on the basis of the unequal distribution of NUMT insertions and on the G+C percentage of each subgenome [21].The CBS 7064 genome contains seven pairs of chromosomes: three are heterozygous, two are homozygous and two are heterozygous along a proportion of their length, indicative of a massive loss of heterozygosity ([21], see Figure S3).Having defined the parental clades of CBS 7064 will allow us to determine which part of the CBS 7064 genome was contributed by which clade.In addition, it was shown that synteny was conserved between the two contributors of CBS 7064, except for a single reciprocal translocation between two chromosomes of one the CBS 7064 contributor [21]. Using the genome sequence of CBS 7064, it was therefore interesting to perform a wider MLST analysis on the typical, haploid strains of the four clades (CBS 185^T^, 2001, 2007 and 2004^T^ from clades 1, 2, 3 and 5, respectively), using 16 markers located at the extremities of each of the chromosomes of CBS 7064 to define the origin of each of the chromosomes of CBS 7064 and to better define each of *M. farinosa* clades.

MLST analysis was obtained for one gene on each extremity of the seven chromosome pairs and on each side of the translocation breakpoints between chromosomes E/F and I/J (Figure S3 and Table S3). Out of the four typical strains tested, only the clade 2 CBS 2001 strain gave 100% sequence identity with at least one of the two alleles of CBS 7064. In the case of the EF-l marker,only 1 bp out of 478 bp differedbetween CBS 7064 and CBS 2001. The three other typical strains: CBS 185^T^ for clade 1, CBS 2007 for clade 3 and CBS 2004^T^ for clade 5 displayed 81 to 96% identity with that of CBS 7064. These results there fore confirmed what we had observed with the *ACT1* and the *RPL33* markers, i.e.a clade 2 strain is a parental contributor of CBS 7064,whileneither clade 1, clade 3 nor clade 5 strains contributed toCBS 7064.Interestingly, none of the strains tested gave 100% identity with the right end markers of chromosomes A and B of CBS 7064 (Figure S3), indicating that the right arm of chromosome A, which is homozygous to the right arm of chromosome B due to LOH [21], corresponds to an as-yet undescribed taxon. Here, we were able to confirm the distinction foundbetween the Pγ and the Pεsubgenomes [21]and to establish unequivocally the source of the parental subgenomes; a clade 2 strain is the contributor of the Pγ subgenome, whereas the Pεsubgenome comes from an as-yet undescribed taxon, which has no haploid representative in our *M. farinose* collection and which we have characterized as clade 4 (Figure 1).

In addition, pair-wise comparisons of the marker sequences in the four typical strains gave identities of 78 to 96%, further supporting the existence of distinct clades within *M. farinosa* based on the 16 chromosomal markers (Table S4). The existence of four divergent sequences for each marker of the typical haploid strains CBS 185^T^, 2001, 2007 and 2004^T^ also indicated thatneither genetic exchange due to hybridization followed by LOH, nor introgression of the tested markers between strains had occurred at the chromosome extremities in representatives of clades 1, 2, 3 and 5.

### Sporulation of the hybrids and characterization of progeny

As some isolates of *M. farinosa* were found to be inter-clade hybrids, it was interesting to examine their ability to sporulate and if they did, the content of the resulting spores. In the genus *Saccharomyces*inter-specific hybrids have been shown to sporulate but to produce unviable spores. In the CTG clade few studies exist on sporulation of pathogen species [5] and none on non-pathogen species, except for CBS 7064, which was described as a sporulating isolate [14].

All the *M. farinosa* inter-clade hybridstrains and a number of haploids listed in Table 1 were allowed to sporulate.All the hybrids sporulated well,exceptfor CBS 4073andhybrid CBS 5949 which did not grow on the sporulation medium used. On the other hand, some of the haploid strains showed little sporulation, if at all; in particular, the typical strains of clade 2 and 3, CBS 2001 and CBS 2007 respectively, did not sporulate, unlike the type strain CBS 185^T^.

The progeny of the clade 2/clade 4 hybrid CBS7064 and of the clade 2/clade 3 hybrid CBS 2006 were analyzed.Only four-spore asci were considered (Figure S4A and [Supplementary-material pone.0035842.s004]S4B). Asci were microdissected and spores were germinated. A total of 52 spores of CBS 7064 out of 163 grew on YPD (32% spore viability). This result seemed surprisingly high for progeny coming from a hybrid of highly diverged parents(15.3% between the two CBS 7064 subgenomes)[21]. Indeed, in *Saccharomyces*, the hybrids with over 10% divergence do not produce viable spores [29]. Using a heterozygous NUMT insertion as marker, we showed that the 52 viable spores had all inherited both parental alleles, suggesting that meiosis had not taken place. Flow cytometry confirmed that 10 of these spores chosen at random had a 2n DNA content (data not shown).

Sporulation of strain CBS 2006 yielded 132 spores, out of which 103 grew on YPD (78% spore viability). A total of 70 spores were analyzedfurther. As the NUMT content of CBS 2006 is not known, we used SNP in genomic markers to analyze the progeny. We amplified and sequenced the AB-r and CD-l markers (which are locatedin CBS 7064 on the right endof the A/B chromosome pair and onthe left endof the C/D chromosome pair, respectively; Figure S3, Table S3). Two spores (designated G6 and 28) carried only one type of CD-l allele, that of clade 3. The rest of the spores carried both clade 2 and clade 3 CD-l alleles. All the spores carried both of the AB-r alleles. This suggested that all the spores except G6 and 28 were diploid, which was confirmed by flow cytometry on 16 spores. Interestingly, the DNA content of spores G6 and 28 were estimated at24.9±0.9 Mb and 13.1±0.2 Mb respectively, the parental CBS 2006 genome size amounting to 21.9±1.5 Mb (Table 2). This suggests a diploid state (∼2.3n)for spore G6 and a haploid or aneuploid state (∼1.23n)for spore 28.

The spores G6 and 28 were further analyzed using theset of 16 markers located on each extremityof all chromosome pairs. The results are summarized in Table 4. Despite a slightly higher number of heterozygous markers than CBS 7064 (9/13 vs. 7/13), the hybrid CBS 2006 harbored also some level of LOH. When compared to its parent CBS 2006, spore G6 contained the same four heterozygous regions at the left and right ends of chromosome pair A/B, left end of E/F and right end of K/L, but homozygous markers were found in the place of the five other heterozygous regions of CBS 2006 (Table 4). Taken together, these results indicate that this spore is diploid and has undergone LOH, possibly during sporulation. On the other hand, spore 28 only displayed a single heterozygous region on the left end of chromosome pair A/B. This spore has a close to 1.23n DNA content with an estimated genome size of 13.1 Mb, which is slightly over the values between 9.2 and 10.8 Mb measured for haploid *M. farinosa* strains. Therefore, spore 28 is probably aneuploid, because it contains both clade 2 and clade 3 AB-r markersbut is homozygous for the other markers and it may have undergone meiosis (Table 4).

**Table 4:** Origin of left and right end side regions of spores G6 and 28 chromosomes.

Markers *	Clades			
	CBS 2006	spore G6	spore 28	CBS 7064
AB-l	2/3	2/3	3	2/4
AB-r	2/3	2/3	2/3	4
CD-l	2/3	3	3	2/4
CD-r	2/3	3	3	2
EF-l	2/3	2/3	2	2/4
EF-r	3	3	3	2/4
GH2-l	2/3	2	2	2
GH2-r	2/3	3	3	2
IJ-l	2	2	2	2/4
IJ-r	2/3	3	na	2/4
KL2-l	3	3	3	2
KL2-r	2/3	2/3	3	2
MN2-l	Na	na	na	2/4
MN2-r	2	2	2	2/4

na: no amplification.

* Several markers from CD chromosomes were also tested (see accession number in Table S2 and marker description in Table S3).

### Analysis of mtDNA origin and NUMT insertions reveals secondary contacts after reproductive isolation

Within *M. farinosa*, we have described distinct clades whose sequence divergence is as high as that described for distinct species, as in the genus *Saccharomyces*for instance. In addition, we have evidenced inter-clade hybridsthat sometimes yield aneuploids. This previously unobserved situation mimics horizontal gene transfer (HGT)and it was interesting to examine potential genetic exchanges in haploid strains. Mitochondria constitute good markers to follow genetic exchanges.

**Figure 2:**
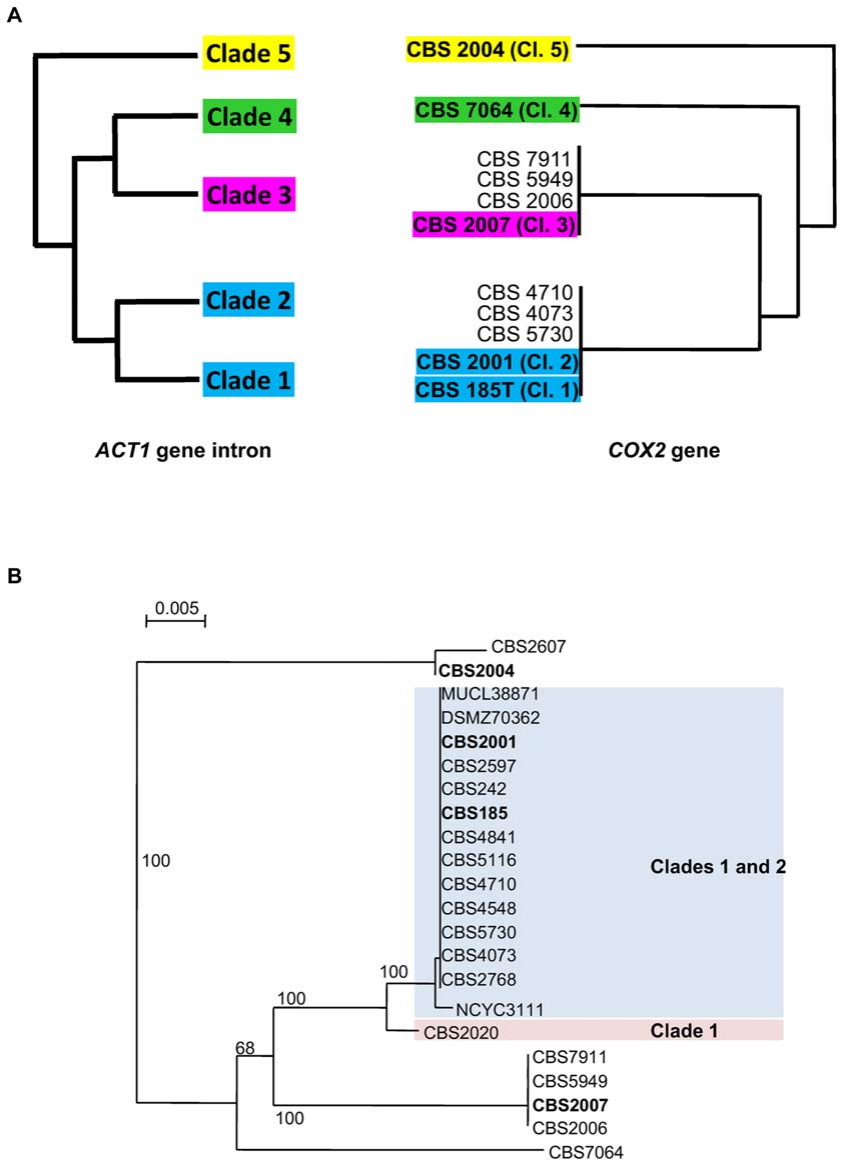
Origin of mtDNA in *M. farinosa* diploid strains. (A) Relation between the clades according to the *ACT1* gene intron tree generated with the sequences of the *M. farinosa* typical strains (left) and to the mitochondrial *COX2* gene of the*M. farinosa* typical strains and of the inter-clade hybrids (right) are shown. Typical strains are in bold characters. Bar, 0.005 substitutions per site. (B) Concatenated *COX2* (540 bp ) and *COX3* (609 bp) gene tree form *M. farinosa* haploid strains of clades 1 and 2. Typical strains are in bold characters. Bar, 0.005 substitutions per site. Tree constructed with *COX2* or *COX3* were congruent.

To address thequestion of the inheritance of mtDNA in the *M.farinosa* inter-clade hybrids, an MLST analysis of the *COX2* and *COX3* genes was first performed using sequence of the part of these two genes in the typical strains of each clade and in all the inter-clade hybrids. A schematic representation of the relationship between the mtDNA sequences in relation with the different clades is shown in Figure 2A. Clade 3 CBS 2007 and clade 5CBS 2004^T^as expectedcarrieddivergentmtDNA alleles, while surprisinglystrains from clade 1 and clade 2 carried the same mtDNA alleles. All the tested hybrids,except CBS 7064 (clade 2/clade 4) and CBS 2167 (clade 3/clade 5), carried either the mtDNA alleles of clade 2 or that of clade 3, consistent with the idea that the hybridswere derived from crosses betweenthe clades. Our results show that, like in *Saccharomyces* [30]inter-specific hybrids [31] or *Metschnikowia* inter-specific hybrids [32], mtDNA can be transmitted by either of the parents in the *M. farinosa* species complex. This is in contrast with several reportson *Saccharomyces*fermentative hybrids involving *S. cerevisiae*showingthat mtDNA from the non-*cerevisiae*parental contributor wasspecifically favored [33].

Interestingly, the mtDNA carried CBS 7064 did not correspond to any of the alleles of clades 1, 2, 3 and 5. Comparison of the mitochondrial genomes of CBS 185^T^ and CBS 7064 had already shown that they differed [18], [19]. From our results, we can deduce that mtDNA of CBS 7064 was transmitted by the parental strain for which we do not yet have a haploid representative strain.

The fact that both clade 1 CBS 185^T^and clade 2 CBS 2001 typical strains carried the same mtDNA alleles prompted us to analyze the distal *COX2* and *COX3* geneson the mitochondrial genome of all haploid strains from clade 1 and 2 in order to try to identify the missing mtDNA type. As represented in Figure 2B, all the stains carried the same mtDNA alleles, except for clade 1 strain CBS 2020, which displayed threeSNP in *COX2*(0.5%) and five SNP in *COX3*(0.8%), demonstratingthat CBS 2020 carried another mtDNA type. These results suggest that mating events between clade 1 and clade 2 ancestors have occurred associated with unidirectional transfer of mtDNA. Unlike in *Metschnikowia* [32], we did not observe any recombination between the mtDNA from different clades.

NUMTinsertions can be found in most yeast genomes [34].Using NUMT insertions that we have identified in the genome of CBS 7064 [21] as markers, we could go further into the analysis of the relationships between the *M. farinosa* strains and confirm the existence of genetic exchanges between clades 1 and 2. By monitoring the presence /absence of NUMT in PCR products, we analyzed nine heterozygous and two homozygous NUMT loci present in the CBS 7064 genome in the three haploid strains CBS 185^T^, (clade 1), CBS 2001 (clade 2) and CBS 2007 (clade 3) as well as and two hybrids CBS 5730 (clade 2/clade 3) and CBS 8045 (clade 2/clade 4). Most insertions were absent in the typical strains from clades 1, 2 and 3, consistent with their presence on chromosomes E, J and M that are characteristic of the clade 4 parent of CBS 7064(Table 5). Conversely, the sole NUMT insertion of chromosome I of CBS 7064, contributed by clade 2 parent, was found in the clade 2 strain CBS 2001 and also in clade 1 strain CBS 185^T^.

**Table 5:** PCR analysis of 11 NUMT loci in six strains of *M. farinosa.*

		NUMT loci											
		heterozygous loci									homozygous loci		
clade	strains	Es9*	Em3	Es12	Es13	Ip	Js18	Js19	Jm4	Ms24	As1	Am1*	
1	CBS 185^T^	−	−	−	−	+	−	−	−	−	−	na	
2	CBS 2001	−	−	−	−	+	−	−	−	−	−	+	
3	CBS 2007	−	−	−	−	na	−	−	−	−	−	+	
2/4	CBS8045	−^a^	−	−	−	+	−	−	−	−	−	+^b^	
2/3	CBS 5730	−^c^	−	−	−	na	−	−	−	−	−	+^b^	
2/4	CBS 7064	+/−	+/−	+/−	+/−	+/−	+/−	+/−	+/−	+/−	+/+	+/+	

The NUMT loci are called with their cognate chromosome letter (upper case) followed by the NUMT number in the sequenced reference strain CBS 7064 [21].

(+) indicates presence of the NUMT; (−) absence of the NUMT; (+/−) both alleles. In CBS 7064, the (+) loci are clade 4, except that on chromosome I which is clade 2.

* indicate that the PCR products were sequenced; ^a^clade 1 sequence; ^b^ clade 2 sequence; ^c^ clade 3 sequence.

na: no amplification.

During the analysis of a NUMT locus on chromosome E (NUMT s9) [21], we discovered that the sequence of the heterozygous allele on chromosome I of CBS 7064 was identical to that of clade 1 CBS 185^T^ and displayed 9% divergence with the sequence of the parentCBS 2001(clade 2), which contributed the rest of the chromosome. Moreover,the clade 2/clade 4 hybrid CBS 8045 (see above) also displayed the clade 1 sequence for this locus. These observations suggest that some genetic exchange has occurred between clades 1 and 2 after reproductive isolation, in agreement with the presence of an identical mtDNA in most strains of clade 1 and clade 2.

### Phylogeny of the M. farinosa complex in the CTG clade and taxonomic considerations

**Figure 3:**
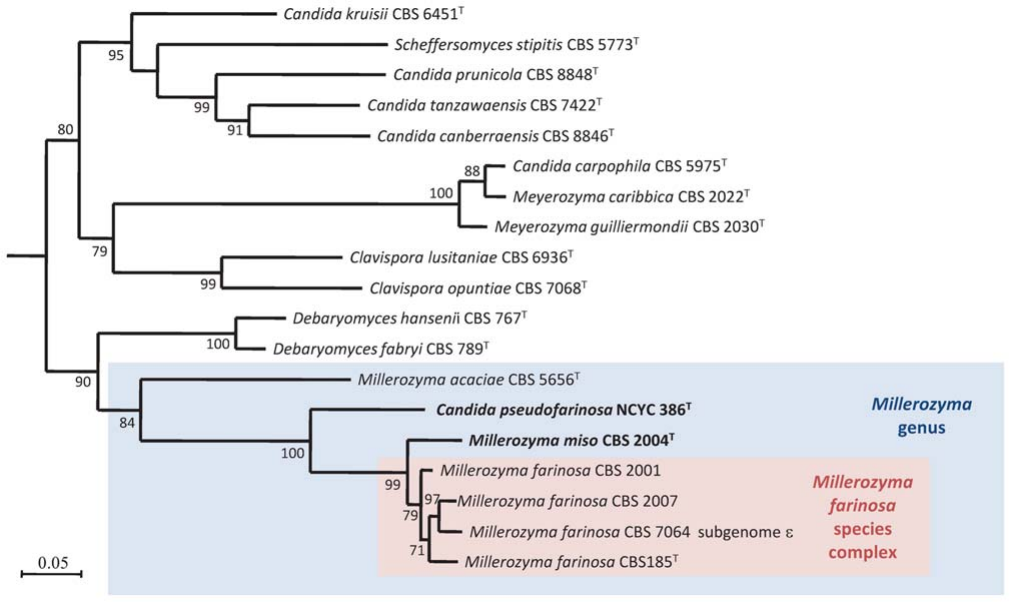
Maximum likelihood tree of the concatenated *ACT1, RPB1* and *RPB2* coding genes of *Millerozyma acaciae*, *Candida pseudofarinosa*, *Millerozyma miso* type strains, of the typical strains of the *M. farinosa*complex and of the most closely related species type strains or strains with a sequenced genome. *Candida fermenticarens* CBS 704^T^ was used as outgroup. Bootstrap values (%) based on 100 replicates are indicated at the nodes. Only bootstrap values over 70% are indicated. Most of the DNA sequences were retrieved from GenBank. Bar, 0.05 substitutions per site.

The *M. farinosa*species was shown to be made of related, but distant clades, and it was therefore necessary to establish a phylogenetic analysis of the species and clades constituting the genus *Millerozyma* based on a reliable multigenic analysis. Kurtzman et al [35]has circumscribed the genus *Pichia*, which now contains the type strain of the genus, *Pichia membranifaciens,* and various species from other previously described genera such as *Issatchenkia*. Kurtzman and Suzuki [9] have renamed the genus to which *P. farinosa* belongs as *Millerozyma*. We generated a phylogeny with the typical strain of each of the four clades, the clade 4 (the Pε subgenome) parent of CBS 7064, NCYC 386^T^,and the type strains of the previously decribed *Millerozyma*species using the *RPB1*, *RPB2* and *ACT1* exon2 sequences (Figure 3). All of the *M. farinosa* typical strains formed a monophyletic group with NCYC 386^T^ having a basal position. We propose that the four closely related clades 1 to 4 that we have defined in this study constitute the species *M. farinosa*. NCYC 386^T^ and CBS 2004^T^ clearly defined two distinct taxa; the high sequence divergence that these two strains display from the other strains of the complex leads us to propose that they can be considered as a new species. We therefore propose to reinstate the species *Zygopichia miso* (type strain CBS 2004^T^ = CLIB 1230^T^) previously used for CBS 2004^T^ by Mogi in 1942 (but which had not been described) under the name *Millerozyma miso* and we would like to define a new species *Candida pseudofarinosa* (type strain NCYC 386^T^ = CLIB 1231^T^).

### Latin diagnosis of *Millerozyma miso* Mogi exMallet, Weiss, Jacques etCasaregola

*In agaro YPD post dies 2 ad 28°C cellulae vegetativae ovoidae aut oblongae, per gemmationem multipolarem reproducentes. Cultura est granula, albida vel cremea, margo glabro vel undulato.In agaro Zea mayidis pseudomycelia formatur.Asci non conjugate et non rumpuntur. Habentes 3–4 ascosporae globosae.*

*Glucosum, galactosum fermentatur. Maltosum, sucrosum, trehalosum, melibiosum, lactosum, cellobiosum, melezitosum et raffinosum non fermentatur.*

*Glucosum, galactosum, d-ribosum, l-rhamnosum, trehalosum, erythritolum, gluconatum et glycerolum assimilantur. Xylosum, sucrosum, maltosum, lactosum, cellobiosum, α-methylum-d-glucosidum, raffinosum,melezitosum, inositolum,acidum lacticum, sorbosum, d-glucosaminum, l-arabinosum, melibiosum, et sodium glucuronatum non assimilantur.Ethylaminum, lysinum et cadaverinum, assimilantur at non nitratum, nitritum creatinum etcreatininum.Vitamina externa crescentia non sunt necessaria. Crescere potest in 10% NaCl et 50% glucosum. 0.01% cycloheximidi non crescit.Maxima temperature crescentiae: 42°C. Materia amyloidea iodophila, acidum aceticum non formantur. Diazonium caeruleum B non respondens. Ureum non hydrolysatur.*

*Typus stirps* CBS 2004^T^( = CLIB 1230^T^)

### Description of *Millerozyma miso* Mogi exMallet, Weiss, Jacques &Casaregola

**Figure 4:**
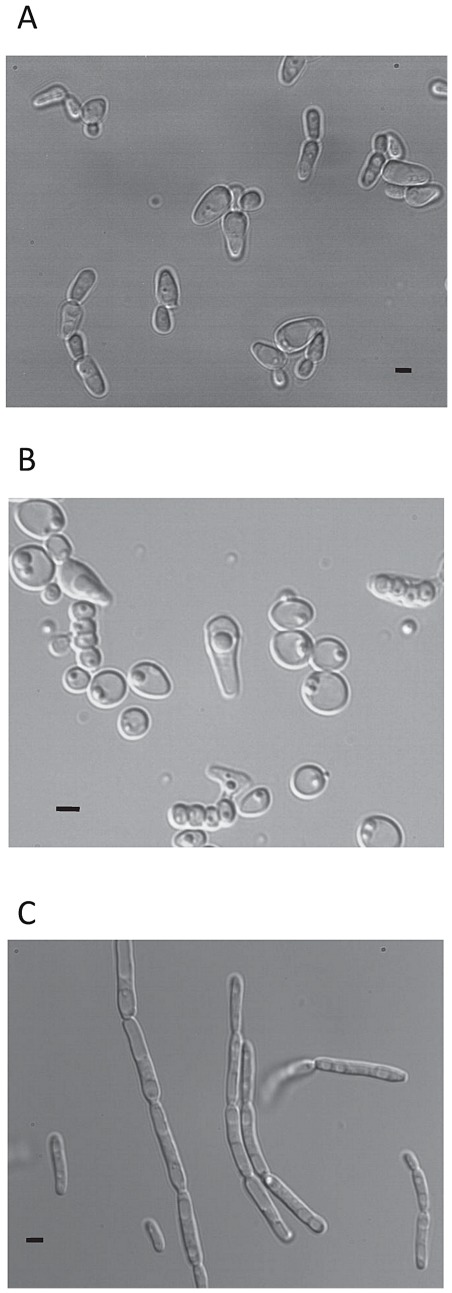
Micrograph of cells. (A): vegetative cells grown on YPD agar for three days at 28°C of *Millerozyma miso* CBS 2004^T^ ; (B) sporulating cells after two weeks on Malt agar at room temperature ofCBS 2004^T^.and (C) vegetative cells grown on YPD agar for three days at 28°C of *Candida pseudofarinosa* sp.nov. NCYC 386^T^.Bar,5 µm.

After 48 h at 28°C on YPD ovoid or oblong vegetative cells are formed (Figure 4A). Cell division is by multipolar budding. After two days on YPD agar the streak is rough, white to cream colored with a slightly undulating margin. Pseudomycelium wasobserved onculture corn meal agar.Formation of ascospores was observed. Asci are persistent and produce 3–4 spherical ascospores (Figure 4B).

*Fermentation:* d-glucose andd-galactose were fermented. d-maltose, saccharose, trehalose, melibiose, lactose, cellobiose, melezitose and raffinose are not fermented.

*Assimilation reaction and other growth tests*: d-glucose, d-galactose, d-ribose, l-rhamnose, trehalose, erythritol, 2-keto-d-gluconate, d-gluconate and glycerol are assimilated. d-xylose, sucrose, d-maltose, d-lactose, d-cellobiose, α-methyl-d-glucoside, d-raffinose, d-melezitose, inositol, d l-lactate, l-sorbose, d-glucosamine, l-arabinose, d-melibiose, sodium glucuronate, are not assimilated. Ethylamine, l-lysine and cadaverine are assimilated; potassium nitrate, sodium nitrite, creatine and creatinine are not assimilated.Growth in vitamin-free medium, 10% NaCl and 50% glucose are positive. 0.01% cycloheximide is negative. Maximum growth temperature is 42°C. Starch-like compounds and acetic acid are not produced. Diazonium blue B reaction is negative. Urease activity is negative.

Synonym: *Zygopichia miso* Mogi, J. Agric. Chem. Soc. Japan 18∶543-554, 629–641, 733–741, 940–944, 1942

Type: CBS 2004^T^( = CLIB 1230^T^)

### Latin diagnosis of *Candida pseudofarinosa* Mallet, Weiss, Jacques et Casaregola sp. nov

*In agaro YPD post dies 6 ad 28°C cellulae vel elongatae pseudohyphae formantes, per gemmationem multipolarem reproducentes. Cultura est butyrosa, sulcuma, centrum sublatum est, albida. In agaro Zea mayidis pseudomycelia formatur.Ascosporae nullae.*

*Glucosum, galactosum, maltosum, sucrosum, trehalosum, melibiosum, lactosum, cellobiosum, melezitosum et raffinosum non fermentatur.*

*Glucosum, gluconatum et galactosum assimilantur. D-ribosum, xylosum, L-rhamnosum, sucrosum, maltosum, lactosum, trehalosum, cellobiosum, alpha-methylum-D-glucosidum, raffinosum, melezitosum, glycerolum, erythritolum, inositolum, sorbitolum, D-gluconatum, acidum lacticum, sorbosum, D-glucosaminum, L-arabinosum, melibiosum, sodium glucuronatum et N-acetyl-glucosaminum non assimilantur.Nitratum, ethylaminum, lysinum, cadaverinum, creatinum et creatininum assimilantur at non nitritum et glucosaminum.Vitamina externa crescentia non sunt necessaria. Crescere potest in 10% NaCl et 50% glucosum. 0.01% cycloheximidi non crescit.Maxima temperature crescentiae: 40°C. Materia amyloidea iodophila, acidum aceticum non formantur. Diazonium caeruleum B non respondens. Ureum non hydrolysatur.*

*Typus stirps* NCYC 386^T^( = CLIB 1231^T^)

### Description of *Candida pseudofarinosa *Mallet, Weiss, Jacques &Casaregolasp. nov

After 6 days at 28°C on YPD, cells are elongated and mycelial pseudohyphae are formed (Figure 4C). Budding is multipolar. After two days on YPD agar the streak is butyrous, wrinkled, moderately raised and white-colored. Pseudomycelium was observed on culture corn meal agar. Formation of ascospores was not observed.

*Fermentation*: D-glucose, D-galactose, D-maltose, saccharose, trehalose, melibiose, lactose, cellobiose, melezitose and raffinose are not fermented.

*Assimilation reaction and other growth tests*: D-glucose, D-gluconate and D-galactose are assimilated. D-ribose, D-xylose, L-rhamnose, sucrose, D-maltose, D-lactose, trehalose, D-cellobiose, alpha-methyl-D-glucoside, D-raffinose, D-melezitose, glycerol, erythritol, inositol, D-sorbitol, 2-keto-D-gluconate, D L-lactate, L-sorbose, D-glucosamine, L-arabinose, D-melibiose, sodium glucuronate, N-acetyl-glucosamine are not assimilated. Potassium nitrate, ethylamine, L-lysine, cadaverine, creatine and creatinine are assimilated; sodium nitrite and glucosamine are not assimilated. Growth in vitamin-free medium, 10% NaCl and 50% glucose are positive. 0.01% cycloheximide is negative. Maximum growth temperature is 40°C. Starch-like compounds and acetic acid are not produced. Diazonium blue B reaction is negative. Urease activity is negative.

The type strain, NCYC 386^T^ ( = CLIB 1231^T^), was isolated in Japan and was deposited at National Collection Yeast Culture by IFO collection during December 1953.

## Discussion

Species whose taxonomy is debatable may reflect the possible existence of a species complex containing cryptic species. By evaluating the sequence divergence in regions little subjected to selective pressure, we have shown that *M. farinosa* was composed of at least four diverged taxa. In addition, two taxa that presented high sequence divergence were considered as new species: *Candida pseudofarinosa* and *Millerozyma miso*. We have previously shown that another CTG species, *D. hansenii*, whose taxonomy has been discussed over the years [36], [37] is a species complex with at least three distinct taxa [23]. Similarly, a third CTG species, *Candida parapsilosis* was also shown to be a complex made of three species [38].

Since the discovery of the inter-specific hybrid lager yeast *S. pastorianus* strains [39], a large number of hybrids have been detected in food [40] and in fermentation environments such as cider making [41], [42], wine making [43], and brewing [44]. As in the *Saccharomyces*, in which hybrids provide new technological potentialitiessuch as specific fermentations [45], hybrids of the CTG clade may form in constrained environmentsand generate new (and beneficial) properties to the cells. In this context, it isperhaps relevant that CBS 7064 and CBS 8045, the sole clade 2/clade 4 hybrids, were both isolated in very high sugar concentrations; the association of genetic material from these two clades may increase resistance to osmotic pressure. However, our test in the laboratory for the growth in high salt concentration (4 M NaCl) did not differentiate these two strains from the other *M. farinosa* isolates (data not shown), in contrast to what was described by [15]; thus new phenotypes associated with hybrid formation in *M. farinosa* remains to be found.

In the *M. farinosa* hybrids, sequence divergence in the *ACT1* intron varied between 8.5 and 14.5%. The overall average genomic divergence between the contributors of CBS 7064 is 15.3% [21], consistent with the value of 15% for the introns of *ACT1* and *RPL33*. Introns are therefore good indicators of overall sequence divergence in yeast genomes, and are superior to sequences like the rDNA D1/D2 [8] or the *ACT1*coding gene [46].

In the *M. farinosa*hybrids, the overall divergence may be reduced by LOH. Indeed, massive LOH was observed in *M*. *farinosa* CBS 7064 to the point where some of the chromosome pairs became entirely homozygous [21]. We confirmed that LOH also occurred at high frequency in other *M. farinosa* hybrids.The distribution of markers in the diploid spore G6showed that the genetic information from one of the parents became predominant. This processseems to vary according to the strain analyzed, as seen from the comparison betweenCBS 7064and spore G6 of CBS 2006. In our study of LOH in a large number of diploids in*D. hansenii,*we observed a tendency, which favored only one parental contributor [47]. The present study demonstrates that *M. farinosa* shares common featureswith other described CTG species such as *C. albicans* [48], *Candidadubliniensis* [49], and *D. hansenii*, for the existence of hybrids and LOH. In addition, natural populations of both haploids and hybrids exist in *M. farinosa* and *D. hansenii*.

In the case of hybrids, LOH would certainly alleviate the heterozygosity-driven inhibition of meiotic recombination. Liti et al [29] have observed a threshold of sequence divergence (around 5%), above which the yield of viable spores in *S. cerevisiae*is greatly diminished. Sequence divergence(as low as 3.6% in populations of *S. paradoxus*) was shown to drastically lower spore viability [50]. The sequence divergence between the *M. farinosa* clades (from 8.5 to 14%) is well above these thresholds and would almost certainly prevent recombination at meiosis if meiosis occurred. Consistent with this observation, our study has shown that there is no shuffling of alleles between analyzed strains using two markers. This suggests that *M. farinosa* has mainly a clonal propagation. Hence, it is possible that the concept of species defined by fecundity as described by Liti et al [29] for the *Saccharomyces* does not apply to yeasts of the CTG clade.

Although most of the *M. farinosa* hybrids did sporulate, one can predict that they do not undergo meiosis. In *Zygosaccharomyces bailii*, a species that is closer to the *Saccharomyces* and does not belong to the CTG clade, a lack of meiotic spores was observed. This was explained by the absence of nuclear fusion after mating [51]. In our study, none of the 52 spores of CBS 7064 and none of the 70 spores of CBS 2006 underwent meiosis, with the possible exception of one spore from CBS 2006 (spore 28). The reason why these yeasts undergo sporulation, but do not perform meiosis is unknown. This is reminiscent of *C. lusitaniae,* whose progeny from crosses was complexand contained 70% haploid spores, 21% diploid spores with the rest being aneuploid [5]. Obviously, the rarity of haploid spores in *M. farinosa* may be due to the important sequence divergence between the parents, which was not the case in *C. lusitaniae*. On the other hand, all the spores from *D. hansenii*inter-clade hybrids are also diploid (our unpublished observation), though in this case the sequence divergence was lower (at most, 3%) than that of *M. farinosa* clades. This observationsuggests that sequence divergence may not be the only reason for the lack of meiosis in these species. Alternatively, in these species, a 3% sequence divergence may still be too high to allow meiosis to occur. It has been proposed that errors in meiosis such as meiosis I non-disjunction or precocious sister segregationor a possibleabsence of synaptonemal complex could lead to aneuploid and diploid sporesin *C. lusitaniae* [5]. Indeed, sporulation of inter-specific hybrids generates aneuploids in *Saccharomyces* [52], because the reduction of meiotic recombination causes a non-disjunction of chromosomes [53]. Therefore, the lack of recombination at meiosis couldexplain the death of aneuploids in *M. farinosa*.

**Figure 5:**
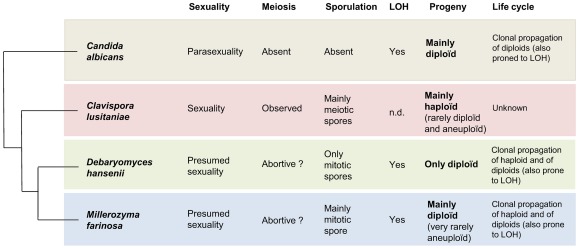
Diversity of the mode of propagation in some yeasts of the CTG clade.nd: non determined.

After sporulation, *M. farinosa* could undergo LOH leading to a reduced heterozygosity allowing meiosis in the resulting organism. The study of the spore G6 confirmed that LOH could be massive in *M. farinosa*, as seen in *M. farinosa* CBS 7064. As in *C. albicans*in which stress affects LOH [54], stress caused by sporulation could increase LOH in *M. farinosa*. Figure 5 summarizes the various situations with regard to meiosis and sexual reproduction in four studied species from the CTG clade. The production of aneuploids by hybrids may be a common feature in the CTG clade, since aneuploids were also described in the species complex *C. parapsilosis* [55]. Interestingly, these authors also observed genetic exchanges in this species that are similar to that which we describe between *M. farinosa*clade 1 and 2.

The progeny of some of the CTG yeast species can therefore be complex, contributing to the diversity in this clade. *M. farinosa* is a mix of haploid clones belonging to different clades, hybrids resulting from mating between these clades and subjected to LOH and aneuploids having variable DNA content resulting from the sporulation of the hybrids through abortive meiosis or possible other mechanisms. The haploid or close to haploid spores resulting from the hybrids therefore display a non-Mendelian marker inheritance. For instance, these haploids may be the ancestor of strains of *M. farinosa* clade 1 or clade 2, which have exchanged material, as deduced from the transmission of mtDNA and from the introgression of unknown extent that we have evidenced from the NUMT analysis. Together, these results indicate that hybridization followedbyLOH is an alternative sexuality for these very divergent strains, although arare occurrence of meiosis isnot excluded. In conclusion, *M*. farinosa constitutes a species complex made of diverged cryptic species, whose genomes are affected by secondary contacts and LOH. We hypothesizethat this situation could be the case for a number of species of the CTG clade. This ensemble of observations invites reflection on thespecies concept in this clade.

## Materials and Methods

### Yeast strains and cultivation

The yeast strains used in this study are from CBS (http://www.CBS.knaw.nl), CECT (http://www.cect.org/), CIRM-Levures (http://www.inra.fr/cirmlevures), DSMZ (http://www.dsmz.de/), MUCL (http://bccm.belspo.be/about/mucl.php), NBRC (http://www.nbrc.nite.go.jp/e/), NCYC (http://www.ncyc.co.uk/), PYCC (http://www.crem.fct.unl.pt/) and VTT (http://culturecollection.vtt.fi/). They are listed in Table 1. Cells were routinely grown in YPD medium (1% yeast extract, 1% peptone, 1% glucose) at 28°C.For sporulation, cells were streaked on sporulation Malt Agar (Difco) plates and were grown for two days at 28°C, then left at room temperature for two weeks. The resulting cells were suspended in 50 µl of zymolyase 100T (1 mg/ml in a 1 M sorbitol solution) for 15 min at 37°C. The 4-spore tetrads were dissected with a Singer MCM System series 200 micromanipulator. Each spore was transplanted on YPD agar plates and incubated at 28°C overnight.

### PCR amplification

The sequences of the nucleotide primersused in this study are listed in Table S1. Primers weredesigned with Primer3 (http://fokker.wi.mit.edu/primer3) on the alignments of genes from complete sequence genomes (http://www.genolevures.org/). Some of the primers were already described [23].Genomic DNA was extracted as previously described [23].

### NUMT analysis

Primers were designed on the complete sequence of M. (*P. sorbitophila*) *farinosa* CBS7064 around each NUMT locus. For the nine heterozygous loci (with the insertion being present on only one parental allele), we chose primer sequences that were identical on both alleles. Two pairs of primers were designed for each of the two homozygous NUMT loci to increase the chance to amplify these regions in all strains.

### Cloning

PCR products were purified using Nucleospin extract II purification kit (Macherey-Nagel, Germany). They were cloned intoa pGemT Easy (Promega, France) using T4 DNA ligase (Promega). DH10B *E. coli* cells were transformed by electroporation with pGemT easy recombinant plasmids. Cells were selected for their ability to grow on 50 µg/ml ampicillin. Positive recombinant clones were chosen after PCR screening for the presence of insert by restriction analysis. Inserts were then sequenced using universal primers (T7 and SP6) or the primers used for the PCR amplification.

### DNA sequence determination and phylogenetic analysis

PCR fragments were sequenced on both strands by Cogenics (Takeley, U.K.) and Eurofins MWG Operon (Ebersberg, Germany). Sequences were assembled with the phred/phrap/consed package. Sequences were analysed with various programs including BLAST implemented at NCBI (http://www.ncbi.nlm.nih.gov). Sequence alignments were generated by using ClustalX [56], MAFFT [57] or Muscle [58] and were manually adjusted with Genedoc (http://www.psc.edu/biomed/genedoc). Phylogenetic trees were reconstructed with Neighbor-Joining program implemented in ClustalX and in MAFFT or with Phyml [59]. Phylogenetic trees were visualized with NJplot [60].

### Flow cytometry

Flow cytometry was performed as described in [47].

### Nucleotide sequence and Mycobank accession numbers

The GenBank/EMBL/DDBJ accession numbers for sequences described in this article are listed in Table S2.The Mycobank (http://www.mycobank.org) accession numbers for *Millerozyma miso* sp. nov. and *Candida pseudofarinosa*sp. nov. are MB 563405 and MB 563406, respectively.

## Supporting Information

Figure S1 Neighbor-joining phylogram of the *RPL33* gene intron of *M. farinosa* isolates. Bootstrap values (%) based on 1000 replicates are indicated at the nodes for main groups and clades. All positions containing gaps and missing data were eliminated from the dataset. Clades are indicated.Typical strains are in bold characters. Heterozygous alleles in hybrids were arbitrarily given the prefix A or B. Bar, 0.02 substitutions per site.
(TIF)

Figure S2 Fluorescence histograms of various *M. farinosa* isolates after propidium iodide staining.
(TIF)

Figure S3 Schematic representation of the localization of the studied markers on the CBS 7064 chromosomes. The representation of the CBS 7064 chromosomes is taken from [21]. Letters on the right indicate the chromosome name. The markers selected for PCR amplification and sequencing are represented by their gene names (Genolevures nomenclature). Simple name used in this study areindicated in red.Orange:clade 2 (Pγ subgenome). Green:divergent sequence from any of the sequences of clades 1, 2, 3 or 5, i.e. clade 4 (Pε subgenome).
(TIF)

Figure S4 Micrograph of sporulating cells after two weeks on Malt agar at room temperature of (A) CBS 7064 and (B) CBS 2006.Bar,5 µm.
(TIF)

Table S1 Primers used in this study.
(DOCX)

Table S2 Accession numbers.
(XLSX)

Table S3 CBS 7064 markers used in this study.
(XLSX)

Table S4 Sequence identity(%) of various markers of the *M. farinosa* typical strains (CBS 185^T^, CBS 2001, CBS 2007) and *M. miso* (CBS 2004^T^) with *M. (P. sorbitophila) farinosa* CBS 7064 and with each other.
(XLSX)
