# Supplementary figures and images for: Insights into the Life Cycle of Yeasts from the CTG Clade Revealed by the Analysis of the Millerozyma (Pichia) farinosa Species Complex
Source: PLoS One. 2012 May 4;7(5):e35842. doi: 10.1371/journal.pone.0035842 (PMC3344839; doi:10.1371/journal.pone.0035842)

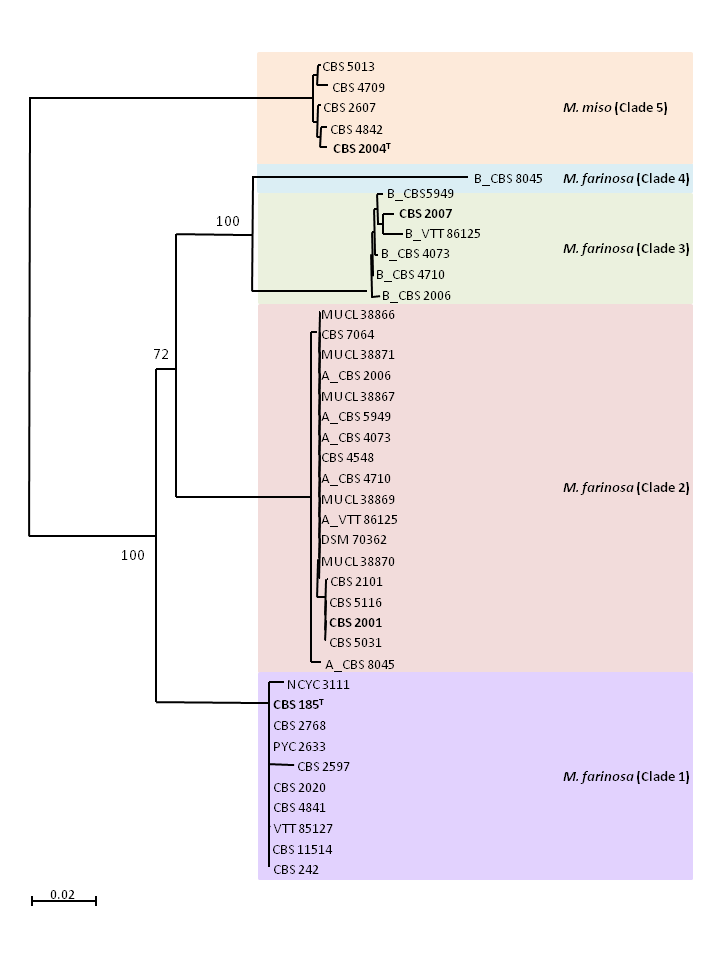

Supplement: Figure S1 — Neighbor-joining phylogram of the RPL33 gene intron of M. farinosa isolates. Bootstrap values (%) based on 1000 replicates are indicated at the nodes for main groups and clades. All positions containing gaps and missing data were eliminated from the dataset. Clades are indicated.Typical strains are in bold characters. Heterozygous alleles in hybrids were arbitrarily given the prefix A or B. Bar, 0.02 substitutions per site. (TIF) [file pone.0035842.s001.tif]

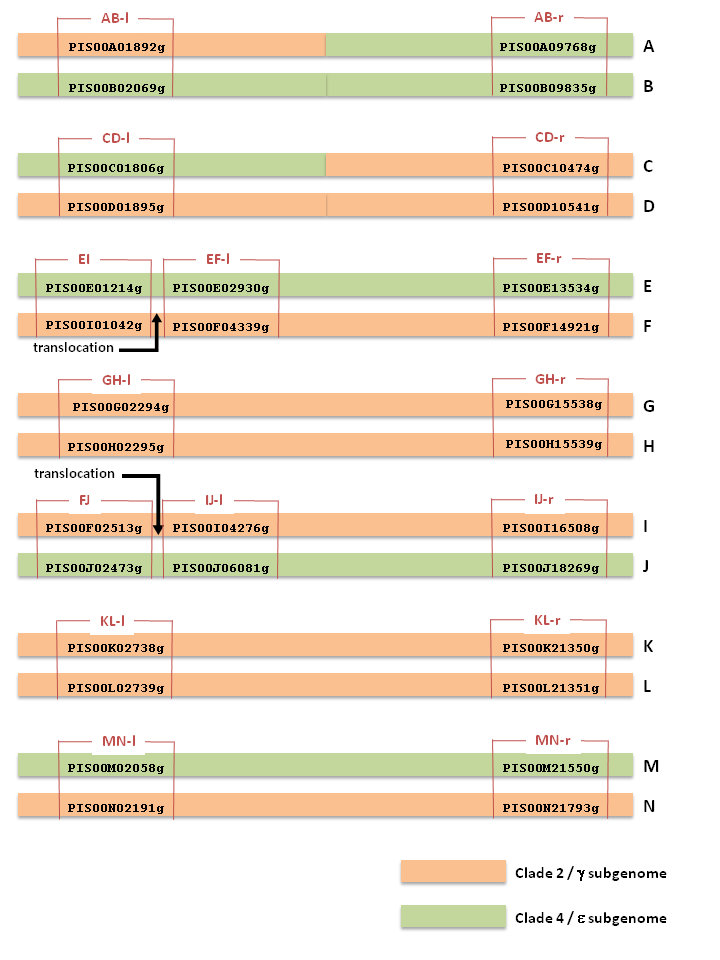

Supplement: Figure S3 — Schematic representation of the localization of the studied markers on the CBS 7064 chromosomes. The representation of the CBS 7064 chromosomes is taken from [21]. Letters on the right indicate the chromosome name. The markers selected for PCR amplification and sequencing are represented by their gene names (Genolevures nomenclature). Simple name used in this study areindicated in red.Orange:clade 2 (Pγ subgenome). Green:divergent sequence from any of the sequences of clades 1, 2, 3 or 5, i.e. clade 4 (Pε subgenome). (TIF) [file pone.0035842.s003.tif]

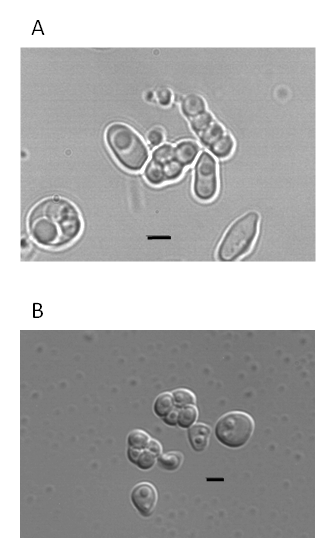

Supplement: Figure S4 — Micrograph of sporulating cells after two weeks on Malt agar at room temperature of (A) CBS 7064 and (B) CBS 2006.Bar,5 µm. (TIF) [file pone.0035842.s004.tif]
