# Supplementary material for: Insights into the Life Cycle of Yeasts from the CTG Clade Revealed by the Analysis of the Millerozyma (Pichia) farinosa Species Complex
Source: PLoS One. 2012 May 4;7(5):e35842. doi: 10.1371/journal.pone.0035842 (PMC3344839; doi:10.1371/journal.pone.0035842)
Supplement: Table S1 — Primers used in this study. (DOCX) [file pone.0035842.s005.docx]

**Table S1**: primers used in this study

| **Target** | **Primer name** | **5’-3’ sequence** | **Reference** |
| --- | --- | --- | --- |
| *ACT1* intron 1 | PISO-AU | ATAGAAAATGGAAGGTATGT | this work |
| *ACT1* intron 1 | PISO-AAL | TGCTCAATAGGGTATCTCAATGTC | this work |
| *ACT1* intron 1 | PISO-ABL | TGCTCAATTGGGTATCTCAATGTC | this work |
| *ACT1* intron 1 | AI2 | AAAAAAAAAAGGAGTATGTAATGG | this work |
| *ACT1* intron 1 | AI3 | TTAAAAAAAATGGAGTATGTAATGG | this work |
| *ACT1* intron 1 | CA2R | CCGTGTTCAATTGGGTATCTCAAGGTC | [[1](#_ENREF_1)] |
| *RPL33* intron 1 | RPL37-U | ATGGCTGAATCACACAGATG | [[2](#_ENREF_2)] |
| *RPL33* intron 1 | RPL37-L | TAAAGAAACGTTTGGGTTGT | [[2](#_ENREF_2)] |
| *ACT1* | CA14 | AACTGGGATGACATGGAGAAGATCTGGGC | [[1](#_ENREF_1)] |
| *ACT1* | CA5R | GTGAACAATGGATGGACCAGATTCGTCG | [[1](#_ENREF_1)] |
| *RPB1* | RPB1_For | ATGCCCAGGTCATTTTGGT | this work |
| *RPB1* | RPB1_Rev | TCAAGTCATCTTCACCTCTGG | this work |
| *RPB2* | RPB2_For | CCAGATGGTGATATTTTGGAAC | this work |
| *RPB2* | RPB2_Rev | CCGATTCAACACTTGAGATACA | this work |
| *RPB2* | RPB2_7R | CCCATWGCYTGCTTMCCCAT | [[3](#_ENREF_3)] |
| *RPB2* | RPB2_F2 | CACCTGAAGGTCAAGCGTGTGG | this work |
| *EFT1* | EFTwo_For | TTGCAAGTCACCAAGGAAGA | this work |
| *EFT1* | EFTwo_Rev | CACGACCGAAAGCGTAGAAT | this work |
| *TEF1* | YTEF_1G | GGTAAGGGTTCTTTCAAGTACGCTTGGG | [[4](#_ENREF_4)] |
| *TEF1* | YTEF_6G | CGTTCTTGGAGTCACCACAGACGTTACCTC | [[4](#_ENREF_4)] |
| *COX2* | CO2PISO_U | GGTATTCATGAATTATATGA | this work |
| *COX2* | CO2PISO_L | CACATTCTAATTTAATAGGCAT | this work |
| ITS | ITS1 | TCCGTAGGTGAACCTGCGG | [[5](#_ENREF_5)] |
| ITS | ITS4 | TCCTCCGCTTATTGATATGC | [[5](#_ENREF_5)] |
| *COX3* | COX3-U | GTCCTTCACCATGACCTATA | this work |
| *COX3* | COX3_L | TATATAAATAATCATATTACA | this work |
| *COX3* | COX3_2U | TATTTACATCATTTAGTTTA | this work |
| *COX3* | COX3_2L | CATCTAATACATGTAGATAT | this work |
| AB-l | AB_G1U | GTGAGTCGTATGAACTACAT | this work |
|  | AB_G1L | ACCCCATGCTAAAGTAGATG | this work |
| AB-r | AB_D3U | GATGACAGAGTTACAGGAAA | this work |
|  | AB_D3L | GTTGTTCAAAACACAGACTT | this work |
| CD-l | CD_G1U | CCTCTGGGCTAACATTGTTAA | this work |
|  | CD_G1L | CTTTCTCATAATCCTGTTAGC | this work |
| CD-r | CD_D1U | ATTTTTCGAAAGTATCATTA | this work |
|  | CD_D1L | ATCTGAGAGTAAAACATCAA | this work |
| EF-l | EF_G1U | GATGAAGATGATGTGGAAGG | this work |
|  | EF_G1L | CATTATAATAACTTCCTCCC | this work |
| EF-r | EF_D1U | TAGTCAGATAAATGAACAAC | this work |
|  | EF_D1L | CTCTAAGTCTTCAGTAGGAT | this work |
| GH-l | GH_G1U | TAGATTTGATAGACTTGTTG | this work |
|  | GH_G1L | TATTAGTGTTAGAGGTAGCA | this work |
| GH-r | GH_D1U | TTGACTATCCTCAATTATTA | this work |
|  | GH_D1L | CTCGATTAAGTTGATTATTA | this work |
| IJ-l | IJ_G1U | TGATTCTGCGGTTGTAAAAC | this work |
|  | IJ_G1L | GAAGCCAACTGAGGGTTAAT | this work |
| IJ-r | IJ_D1U | GAAGCTGGAATTGAATGTAA | this work |
|  | IJ_D1L | CAGATATGAAGTTGGTACCT | this work |
| KL-l | KL_G4U | TGGACGTTATAGAACTAGGA | this work |
|  | KL_G4L | CTAACAAGAAATGCTGTCTC | this work |
| KL-r | KL_D1U | ATGGCTGAATCACACAGATG | this work |
|  | KL_D1L | TAAAGAAACGTTTGGGTTGT | this work |
| MN-l | MN_G1U | GAGTAAGTCAAAGGCAGAAC | this work |
|  | MN_G1L | TCTTGAGAGCATTGGAGGTG | this work |
| MN-r | MN_D1U | ATGCCCAGGTCATTTTGGT | this work |
|  | MN_D1L | TCAAGTCATCTTCACCTCTGG | this work |
| EI | EI_1U | GGAAAACGAGGAAGATATGA | this work |
|  | EI_1L | TTTGCTTCTTCCTCTACCAT | this work |
| FJ | FJ_1U | GATGATTTTGACACCTTTGA | this work |
|  | FJ_1L | TATCCTCTCAACCCGAATCT | this work |
| GH-l | GHG2-U | CTCCACAAATGAAGACATAC | this work |
|  | GHG2-L | TCTCTAGAGTCAATCAACCA | this work |
| GH-r | GHD2-U | GCTTTTCAGCTTATTGAGGA | this work |
|  | GHD2-L | TTTCCTAATCTACGGGTCAA | this work |
| KL-l | KLG2-U | TCGGTAGAGGTCAAAGAGAA | this work |
|  | KLG2-L | TCCAAGAAGATTTGACCATC | this work |
| KL-r | KLD2-U | GTATATGTTAAGGGCAAGCAC | this work |
|  | KLD2-L | AGATGTTAGATGGGTACAACA | this work |
| MN-l | MNG2-U | TTCTGGGGTTTAGACAAAAA | this work |
|  | MNG2-L | TTCAGCTAATTTGGACCATT | this work |
| MN-r | MND2-U | TCTGTTGTTAGAGTTTTGGT | this work |
|  | MND2-L | TCTTTCTAACACCTGGAATA | this work |
| CD1 | CD1-U | TGCTGTTCTTTTACTTATAG | this work |
|  | CD1-L | ATACGTATATTTGATGTGAA | this work |
| CD2 | CD2-U | CTTTATGGCGGGTTCTTTGA | this work |
|  | CD2-L | GAGCTTCCATTGACGGTTTC | this work |
| CD3 | CD3-U | CCAAAAGAGCGGAAGAACAG | this work |
|  | CD3-L | ATTTCCGATTGATCCCCTTC | this work |
| CD4 | CD4int-U | ACTCTCAAACTGAACAAGTG | this work |
|  | CD4int-L | GAAATGATTGTTCCTTACG | this work |
| CD5 | CD5bisint-U | AAGTTGGGTGTCTTAATCTA | this work |
|  | CD5bisint-L | TCCAACAATTCTAATAAAGC | this work |
| CD6 | CD6-U | CTCTTTATTGATTGATTTGG | this work |
|  | CD6-L | GAAGCTATATGCTTTCTTGA | this work |
| CD7 | CD7-U | GAGACGAGCTCCATAAGTCG | this work |
|  | CD7-L | GGGATACACTTGTAAGTCA | this work |

1. Daniel HM, Meyer W (2003) Evaluation of ribosomal RNA and actin gene sequences for the identification of ascomycetous yeasts. Int J Food Microbiol 86: 61-78.

2. Jacques N, Mallet S, Casaregola S (2009) Delimitation of the species of the *Debaryomyces hansenii* complex by intron sequence analysis. Int J Syst Evol Microbiol 59: 1242-1251.

3. Liu YJ, Whelen S, Hall BD (1999) Phylogenetic relationships among ascomycetes: evidence from an RNA polymerse II subunit. Mol Biol Evol 16: 1799-1808.

4. Kurtzman CP (2003) Phylogenetic circumscription of *Saccharomyces*, *Kluyveromyces* and other members of the Saccharomycetaceae, and the proposal of the new genera *Lachancea*, *Nakaseomyces*, *Naumovia*, *Vanderwaltozyma* and *Zygotorulaspora*. FEMS Yeast Res 4: 233-245.

5. White TJ, Bruns T, J T (1990) Amplification and direct sequencing of fungal ribosomal RNA genes for phylogenetics. In: Innis MA, Gelfand DH, Sninsky J, White TJ, editors. PCR protocols a guide to methods and applications. San Diego: Academic Press. pp. 315-322.
